# Cation exchange chromatography removes FXIa from a 10% intravenous immunoglobulin preparation

**DOI:** 10.3389/fcvm.2023.1253177

**Published:** 2023-11-22

**Authors:** Gil Bu Kang, Alan Huber, Jihye Lee, Min-Jung Kim, Eun Bang, Jeungwoon Hong, Seulgi Park

**Affiliations:** ^1^ R&D Center, GC Biopharma Corp., Yongin-si, Republic of Korea; ^2^ Department of Medical Affairs, GC Biopharma USA, Inc., Fort Lee, NJ, United States

**Keywords:** intravenous immune globulin, thromboembolic event, coagulation FXIa, procoagulant activity, cation exchange chromatography

## Abstract

The presence of residual activated coagulation factor XI (FXIa) in some commercial intravenous immunoglobulin (IVIG) products has been identified as the root cause of a small number of thromboembolic events in patients who had received such therapy. Our objectives here were to design and evaluate the manufacturing process of GC5107, a 10% glycine-stabilized IVIG product, for its capacity to remove FXIa. The manufacturing process included a cation exchange chromatography (CEX) step, which employs a resin that binds immunoglobulin G (IgG) with high capacity. Procoagulant activity was assessed using Western blot analysis, enzyme-linked immunosorbent assay, thrombin generation assay, chromogenic FXIa assay, and non-activated partial thromboplastin time (NaPTT) assay. A spiking study in which large quantities of FXIa were added to samples before CEX chromatography was used to examine the robustness of the process to remove FXIa. Western blot and ELISA analyses demonstrated that residual FXIa remained in the intermediate manufacturing products until after CEX chromatography, when it was reduced to undetectable levels. The spiking study demonstrated that CEX chromatography removed >99% of FXI protein and reduced FXI activity to below detection limits, even in samples containing 158-fold greater FXIa levels than that of normal samples. Procoagulant activity in 9 consecutive lots of GC5107 was reduced to below the detection limits of the thrombin generation and chromogenic FXIa assays (<1.56 IU/ml and <0.16 IU/ml, respectively). The NaPTT of >250 s in all 9 lots indicated very low levels of procoagulant activity. We demonstrate that a novel 10% IVIG manufacturing process including CEX chromatography is a robust means of removing FXIa from the final preparation.

## Introduction

Immunoglobulin (IG) therapy is a mainstay of treatment for patients with primary immunodeficiencies (1). There are numerous licensed IG products available, formulated for either intravenous (IV) or subcutaneous administration. While all human IG is manufactured from pooled plasma using variations on the Cohn–Oncley cold ethanol fractionation process, differences in starting material, downstream purification steps and added stabilizers result in final products that differ in composition in ways that may be important for individual patients (2–4).

Published reports of a small number of thromboembolic events (TEEs) occurring in patients receiving high doses of IVIG in the 1990s were attributed to an increase in blood viscosity following infusion, or to patient-related factors such as heart disease, renal insufficiency or prolonged immobilization (5–7). Subsequent investigations, however, identified activated coagulation factor XI (FXIa) as a contaminant in some commercial IG preparations (8, 9). In 2010, an unexpected increase in TEEs associated with use of an IVIG product resulted in its temporary removal from the market—residual FXIa was subsequently confirmed to be the root cause (10).

FXI is a zymogen that plays a key role in activation of the intrinsic coagulation pathway by activating its primary substrate, factor IX (FIX). It can also activate additional coagulation factors (11, 12). It has been shown that even small quantities of FXIa can result in significant thrombin generation (9). Regulatory agencies have provided guidance for manufacturers to assess the presence of FXI in IG preparations, and a Boxed Warning for all IG products for risk of thrombosis was mandated by the FDA in 2013 (13). Recognizing that patients with thrombotic co-morbidities are at particular risk, the warning includes precautions for IG administration in these patients.

Human IgG has an isoelectric point (pI) ranging from 6.4 to 9.0, while the pI for FXIa ranges from 8.9 to 9.1 (14). Because of this similarity, is difficult to separate FXIa from IG preparations using ethanol precipitation alone. Additional purification methods used by manufacturers to separate IgG from FXIa have included a combination of octanoic acid (caprylic acid) precipitation or various chromatographic methods (15, 16). FXIa has been characterized as a Critical Quality Attribute in process optimization (17), emphasizing the importance of FXIa removal as its persistence in intermediary products can risk the presence of procoagulant activity in the final IG preparation. The development of additional, robust manufacturing processes that remove residual FXI from IVIG remains an important goal, with the aim of further improving product safety.

We have previously described a novel manufacturing process for a 5% maltose-stabilized IVIG product (GC5101) and successfully demonstrated its capacity to remove FXIa (18). GC5107 is a 10% glycine-stabilized IVIG produced by GC Biopharma. Results of the phase 3 clinical trial in patients with primary immunodeficiency have been published previously (19). Here, we describe in detail the manufacturing process for GC5107 and demonstrate that cation exchange (CEX) chromatography is a robust means of removing FXIa from the final preparation, even in starting material spiked with high levels of FXIa.

## Materials and methods

### Study design and manufacturing process

**Figure 1:**
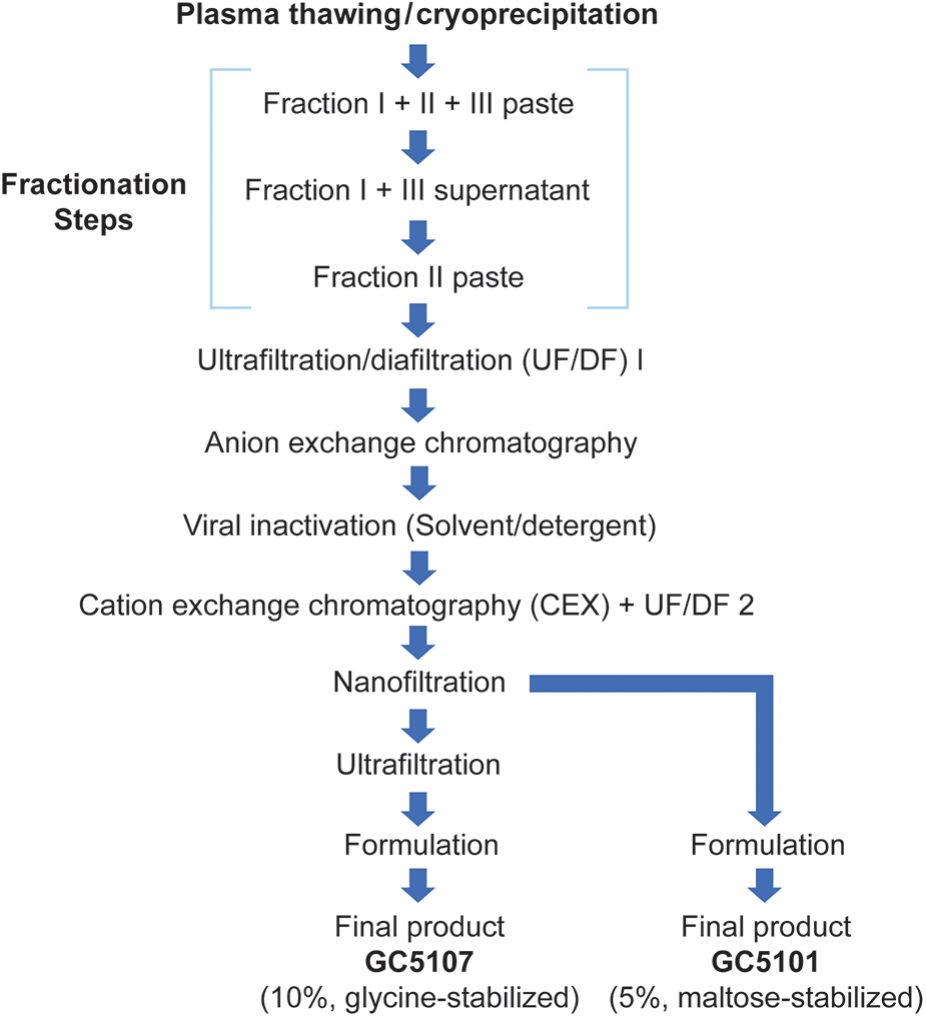
Flowchart of the GC5107 and GC5101 manufacturing processes.

A flow diagram of both the GC5107 and GC5101 manufacturing processes are shown in Figure 1. The processes are identical up to and including the nanofiltration step. For both products, cryo-poor pooled plasma was used as the starting material. Cold ethanol fractionation produced a paste containing fraction I + II + III, a supernatant containing fraction I + III, and a fraction II paste. The fraction II paste was dissolved in 0.6% sodium chloride solution, the pH was adjusted to 5.0, and the solution subjected to clarifying depth filtration (ultrafiltration/diafiltration, nominal size: 0.1 mm). Then 1 M sodium acetate was added to the diafiltrated IgG solution to a final concentration of 5.0 mM, the pH was adjusted to 6.0, and the solution was subjected to anion exchange (AEX) chromatography. The unbound fraction was collected, the pH was adjusted to 5.0 and then subjected to the first viral elimination step, solvent/detergent (S/D) treatment. Tri(n-butyl)-phosphate (TNBP) and polysorbate 80 (Tween 80) were added at concentrations of 0.3% and 1.0%, respectively, and the mixture was incubated for 8 h at 25°C. Sodium acetate 1 M was added to the filtrate, which was then subjected to an additional clarifying depth filtration just prior to CEX chromatography to absorb IgG. Following CEX chromatography, the filtrate was washed with equilibrium buffer (20 mM sodium acetate, pH 5.0) and eluted with elution buffer (20 mM sodium acetate with 0.5 M sodium chloride, pH 4.5) to acquire IgG. The salt content was eliminated by ultra/diafiltration (UF/DF). Nanofiltration (Ultipor®VF DV 20, Pall Lifescience, Switzerland) was then performed as the second viral elimination step. Following nanofiltration, in contrast to GC5101, GC5107 undergoes an additional ultrafiltration step prior to formulation. For GC5107 the nanofiltration solution was concentrated using a Millipore Pellicon 2 cassette. Glycine was added to the ultrafiltration solution as a stabilizer prior to adjusting protein concentration and pH. The final product was obtained following sterile filtration. Procoagulant activities were assessed in each of the intermediate and final products.

### Spiking study

For the spiking study, commercial FXIa [Hematologic Technologies Industries (HTI), USA] was added to the pre-CEX intermediate products (post-S/D treatment) at concentrations of 2.8 µg/ml and 14.0 µg/ml. FXI content was assessed in the pre- and post-CEX products using the Human Total FXI ELISA Kit (Innovative Research, USA).

### Assays used for FXI/FXIa assessment

FXI/FXIa content in the intermediate and final products was analyzed using Western blot analysis and enzyme-linked immunosorbent assay (ELISA). Procoagulant activity in the final product was analyzed using the thrombin generation assay (TGA), chromogenic FXIa assay, and NaPTT assay. Each method was verified based on ICH Guideline Q2 (R1) (20).

### Western blot analyses and ELISA

For Western blot analysis, samples were subject to sodium dodecyl sulfate-polyacrylamide gel electrophoresis (SDS-PAGE) and electroblotted onto a polyvinylidene difluoride (PVDF) membrane by semi-dry transfer (Bio-Rad, USA). Following overnight blocking at 4°C, primary antibodies were applied. Antibodies included anti-prothrombin [factor II (FII)] mouse monoclonal IgG (Merck), anti-factor VII (FVII) rabbit monoclonal IgG (Abcam), anti-factor IX (FIX) mouse monoclonal IgG (Santa Cruz Biotechnology, USA), anti-factor X (FX) rabbit polyclonal IgG (Abcam), and anti-FXI antibody provided in the Human Total FXI ELISA Kit (Innovative Research, USA). Horseradish peroxidase-conjugated secondary antibodies (Innovative Research, USA), were incubated for 1 h and signals were detected with chemiluminescence reagents (Intron, USA). Band density of FXI/FXIa was calculated using Image Lab 5.2.

ELISA assays were performed using the Human Total FXI ELISA Kit (Innovative Research, USA).

### Thrombin generation assay (TGA)

The TGA was conducted in accordance with the CBER Ig-Thrombin Generation Test Protocol (automated version) (21). The final reaction volume consisted of 50% factor XI-deficient plasma (HTI, USA), 1.25% 64 mM substrate (Bachem, Switzerland), 2.5% 0.5 mM phospholipid (Rossix, Sweden), 2.5% 6 mM tissue factor (Dade Innovin®, USA), 8.75% 114.5 mM CaCl_2_ (Sigma Aldrich, USA), and 35% diluted test sample. FXIa product [National Institute of Biological Standards and Control (NIBSC), 13/100] was diluted to concentrations of 5.0, 2.5, 1.25, 0.625, 0.313, and 0.156 mIU/ml as the standard curve. Standard FXIa was spiked into a sample as a positive control.

Activated thrombin in the test samples was measured at 37°C for 1 h at 40-second intervals using a fluorescent reader (Infinite F-500, Tecan, Switzerland; Ex: 380 nm, Em: 430 nm). The maximum thrombin value was calculated using the Origin Program. Then, a 4-parameter curve was plotted using the Softmax program with the concentration of the standard solution and the maximum thrombin value of the standard solution. The FXIa content of the sample is an average value converted from each dilution factor (duplicated at 3 concentrations for each sample). The limit of detection is 1.56 mIU/ml, confirmed from analytical method validation.

### Chromogenic FXIa

FXIa functional activity in the final product was measured using the Rox Factor XIa kit (Rossix, Sweden). Samples were analyzed according to the kit instructions. In this assay, FXIa present in the test sample activates FIX to FIXa in the presence of calcium ions (CaCl_2_). Generated FIXa then activates FX (FXa) in the presence of FVIII, phospholipids and CaCl_2_. FXa then hydrolyzes a chromogenic substrate (Z-D-Arg-Gly-Arg-pNA). The resulting product (p-nitroaniline) was measured at a wavelength of 405 nm (reference wavelength: 490 nm). The FXIa calibrator, a kit component, was used as the standard. Results shown represent one assay per sample and each assay was run in duplicate. There were no controls in the assay. The assay used the spiking method for suitable measurement of FXIa.

### NaPTT

The NaPTT assay was used to indicate the presence of activated coagulation factors in the final product and was performed according to the European Pharmacopeia 7.0 (22). Samples were diluted 1:5 and 1:10 in Tris-albumin buffer. Tris-albumin buffer and standard- (Factor XIa, NIBSC) spiked Tris-albumin buffer were used as negative and positive controls, respectively. Diluted samples (or controls) were added to tubes containing platelet-poor plasma (Technoclone, Austria) and FXI-deficient plasma (HTI, USA), followed by phospholipid diluted 250-fold (0.25 mM into 1 µM) (Rossix, Sweden) and CaCl_2_. Clotting time (time that elapsed between the addition of CaCl_2_ and the formation of a clot) was measured using an automated coagulator (ACL-TOP 500, Werfen, USA). The test was considered valid if the clotting time of the negative control was 200–350 s (as suggested by the European Pharmacopeia), the clotting time of the positive control with platelet-poor plasma was <200 s (set for system suitability in the assay), and the negative control with FXI-deficient plasma was >200 s. Minimal and discriminating FXIa concentration of the positive control was determined (100 mIU/ml).

## Results

### CEX reduces FXI/FXIa to below detection limits

**Figure 2:**
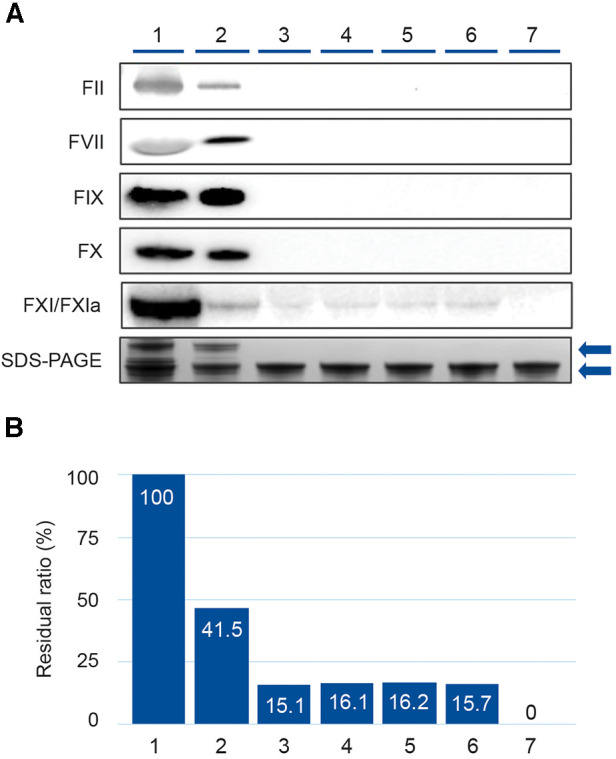
Western blot analysis demonstrating removal of coagulation factors (FII, FVII, FIX, FX, and FXI/FXIa) during the GC5107 manufacturing process. (1) Cryo-poor plasma, (2) Fraction I + II + III paste, (3) Fraction I + III filtrate, (4) Fraction II paste resuspension, (5) Ultrafiltration/diafiltration (UF/DF) 1 solution, (6) Anion exchange chromatography (AEX) flow through, (7) Cation exchange chromatography (CEX)+UF/DF 2 solution. (**A**) Image of Western blot of coagulation factors with SDS-PAGE loading controls. Gels were 4%–12% Bis-Tris (FII, FVII, FIX and FX) or 3%–8% Tris-acetate (FXI/FXIa). Loading amount was 22.5 µg IgG. SDS-PAGE loading amount was 5.0 µg IgG. Upper arrow: IgG polymer, lower arrow: IgG monomer. (**B**) Band densities of the FXI/XIa Western blots were calculated using Image Lab 5.2.1 and are expressed as % residual ratio relative to cryo-poor plasma.

Results of the Western blot analysis performed on intermediate and final products are shown in Figure 2. While FII, FVII, FIX and FX were removed during fractionation (lanes 3–7), residual FXI/FXIa persisted in the fractionation products after AEX but was reduced to undetectable levels by CEX chromatography (Figure 2A). Band intensity analysis of the FXI/FXIa Western blot revealed that residual FXI/FXIa remained present at approximately 16% residual ratio in the intermediate products until after CEX chromatography, when it was reduced to undetectable levels (Figure 2B).

**Figure 3:**
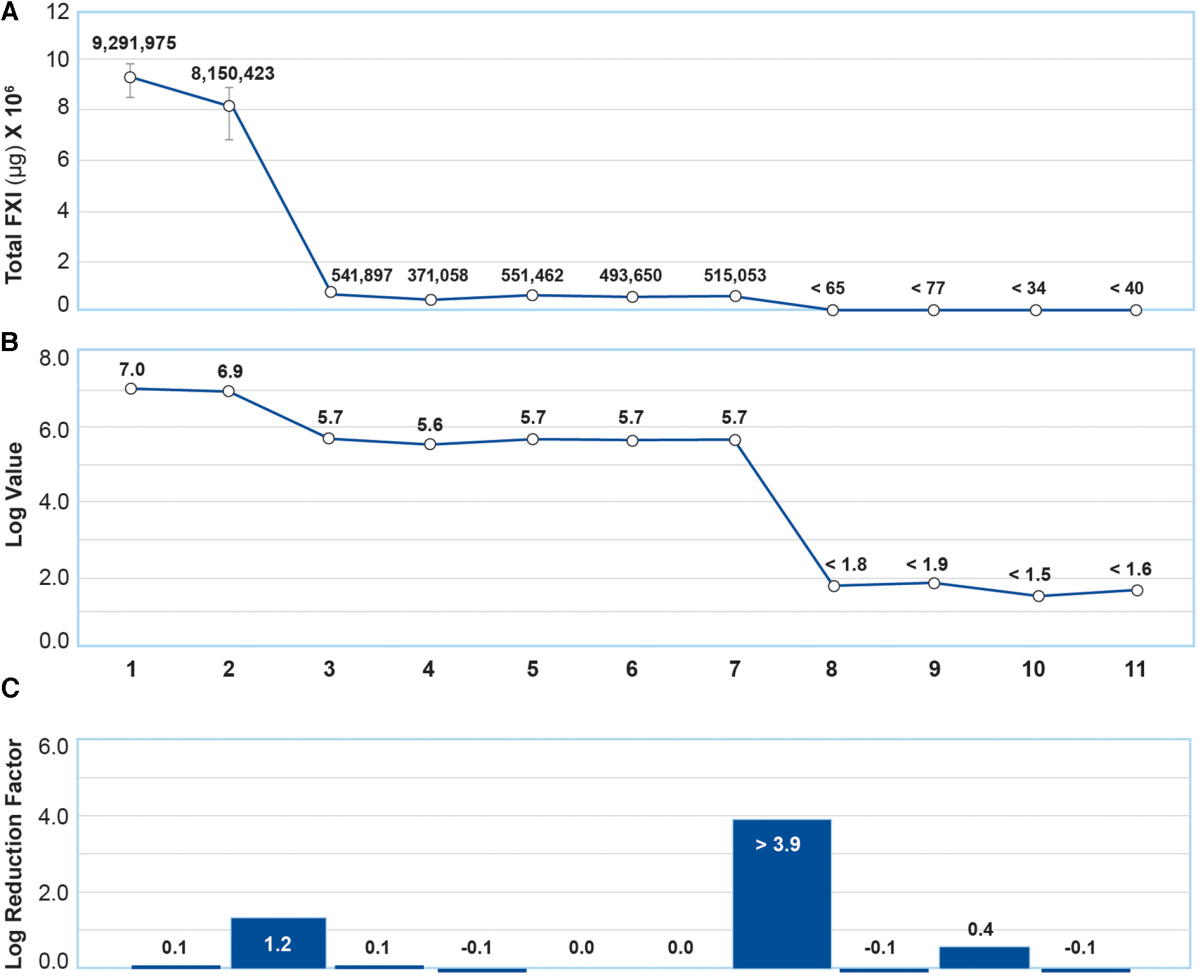
Analysis of total FXI content using ELISA. (**A**) Total FXI (µg/ml) in each intermediate product. Data are mean ± SD of 3 manufacturing batches. (**B**) Total FXI results in (**A**) were transformed to obtain log values. (**C**) FXI log reduction factors reflected in (**B**) (1) Cryo-poor plasma, (2) Fraction I + II + III paste resuspension, (3) Fraction I + III supernatant, (4) Fraction II paste resuspension, (5) Ultrafiltration/diafiltration (UF/DF) 1 solution, (6) Anion exchange chromatography (AEX) flow through, (7) Solvent/Detergent treatment, (8) Cation exchange chromatography (CEX)+UF/DF 2 solution (9) Nanofiltration product, (10) Ultrafiltration product, (11) Final product. The entire GC5107 manufacturing process resulted in a FXI log reduction factor of ≥5.4.

Total FXI levels in each intermediate product in 3 manufacturing lots were analyzed using ELISA. The results are shown in Figure 3A. The FXI log reduction factor at the fractionation I + III step was 1.2, and ≥3.9 at the CEX + UF/DF 2 step (Figures 3B,C). The entire GC5107 manufacturing process resulted in a FXI log reduction factor of ≥5.4. Following the CEX + UF/DF 2 step, the FXI level was less than the limits of quantification.

### Verifying capacity for FXI removal

**Figure 4:**
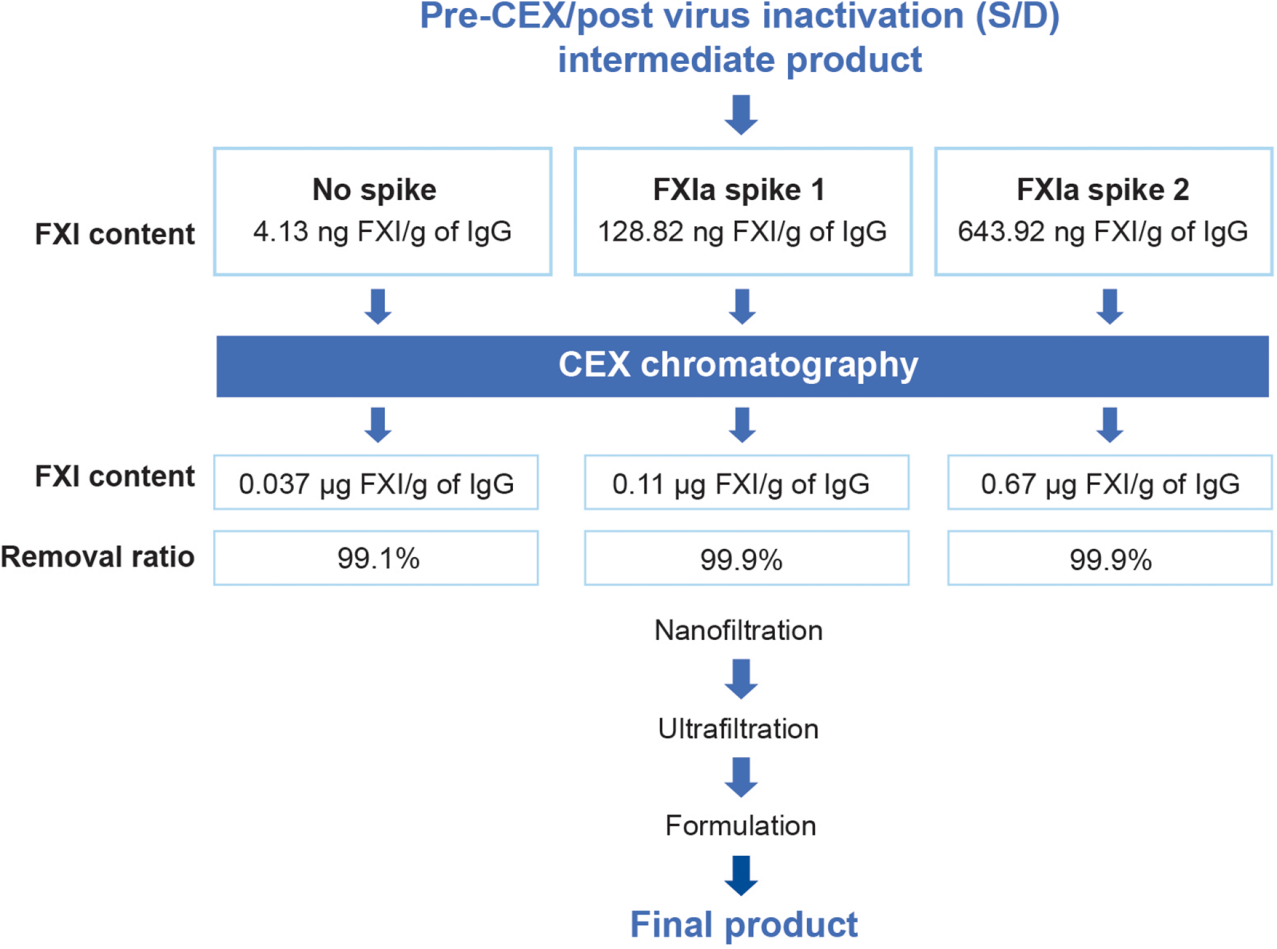
Spiking study flowchart. To validate the ability of CEX chromatography to remove FXIa, commercial FXIa was added to the pre-CEX intermediates (post virus inactivation) at concentrations of 2.8 µg/ml (spike 1) and 14.0 µg/ml (spike 2). FXI content was assessed by ELISA in both the pre-CEX intermediates and the post-CEX eluate. FXI content is expressed as µg FXI/g of IgG. The added FXIa resulted in FXIa concentrations that were 32.0 times and 158.3 times higher than those of normal samples.

Because starting plasma can vary in FXIa content, a spiking study was performed to verify the capacity of the GC5107 manufacturing process to remove even large quantities of FXIa. For this study, commercially available FXIa was added to the pre-CEX, post-viral inactivation intermediate product as shown in Figure 4. The average FXI/FXIa concentration of the cryo-poor plasma samples at the start of the GC5107 manufacturing process was approximately 2.8 µg/ml (data not shown). Addition of commercial FXIa at concentrations of 2.8 µg/ml and 14.0 µg/ml (approximately 5 times the concentration in the starting cryo-poor plasma) represents 32.0 times and 158.3 times higher concentrations than that of normal samples, respectively. Figure 4 shows that CEX chromatography was capable of removing >99% of FXI, even in samples containing 158 times greater FXI levels than that of normal samples.

Levels of FXI/FXIa protein concentration and FXIa activity before and after CEX chromatography are shown in Table 1. The concentration of FXI/FXIa protein that remained after CEX chromatography was low, even in the sample spiked with FXIa at levels more than 150 times the amount present in normal specimens. FXI residual ratio calculations were performed to measure the efficiency of the process for removing FXIa. These demonstrated that only 0.1% of the FXI present prior to CEX chromatography remained in the product after this step (Table 1).

**Table 1:** FXI antigen and activity following CEX chromatography.

	No spike		FXIa spike 1 (2.8 µg/ml)		FXIa spike 2 (14.0 µg/ml)	
	Pre-CEX	Post-CEX	Pre-CEX	Post-CEX	Pre-CEX	Post-CEX
FXI/XIa protein (ELISA^a^), ng/ml	83.79	<0.31	2,679.48	0.96	13,264.79	5.56
FXIa activity (TGA^b^), mIU/ml	22.26	<1.56	73,159.54	<1.56	367,549.59	<1.56
IgG recovery (%)	103.3		100.8		102.4	
FXI residual ratio (%)	0.9		0.1		0.1	

ELISA and TGA were analyzed in duplicate.

^a^ ELISA, enzyme-linked immunosorbent assay.

^b^ TGA, thrombin generation assay.

### Assessment of procoagulant activity

In order to determine whether FXIa activity remained after CEX chromatography, procoagulant activity in the pre-and post-CEX samples was assessed using TGA assays. Though procoagulant activity was present in all pre-CEX samples (at extremely high levels in the spiked samples), it was reduced to below detection limits after CEX chromatography, even in the sample spiked with more than 150 times the amount of FXIa present in normal samples (Table 1). The CEX chromatography step had no effect on IgG recovery.

Nine consecutive batches of GC5107 were produced on a commercial scale using the process described in Figure 1. In order to gauge the potential for GC5107 to induce thrombogenic events, procoagulant activity in the final products was assessed using the TGA, chromogenic FXIa and NaPTT assays. These results are shown in Table 2. The results of the TGA and chromogenic FXIa assays in all 9 batches were below the detection limits (<1.56 mIU/ml and <0.16 mIU/ml, respectively). The coagulation time measured using the NaPTT assay was >250 s in all batches and were thus considered to have no procoagulant activity.

**Table 2:** Assay results of final products.

Batch no.^a^	TGA^b^ (mIU/ml)	Chromogenic FXIa (mIU/ml)	NaPTT^c^ (s)			
			1:5	Ratio^d^	1:10	Ratio^e^
1	<1.56	<0.16	267.0	1.0	266.2	1.0
2	<1.56	<0.16	282.6	1.1	268.8	1.1
3	<1.56	<0.16	268.1	1.0	275.6	1.1
4	<1.56	<0.16	299.4	1.0	291.9	1.0
5	<1.56	<0.16	302.4	1.0	304.8	1.0
6	<1.56	<0.16	300.8	1.0	296.6	1.0
7	<1.56	<0.16	265.9	1.1	265.9	1.1
8	<1.56	<0.16	256.8	1.1	259.6	1.0
9	<1.56	<0.16	271.3	1.1	266.7	1.1
Mean ± SD	<1.56	<0.16	279.4 ± 17.5	1.0 ± 0.1	277.3 ± 16.2	1.0 ± 0.1

Results of assays of the final products of 9 consecutive batches of GC5107.

^a^ Data from 9 batches after sterile filtration and maltose addition.

^b^ TGA, thrombin generation assay.

^c^ NaPTT, non-activated partial thromboplastin time.

^d^ Ratio of the clotting time of the sample diluted 5 times to the clotting time of the control buffer.

^e^ Ratio of the clotting time of the sample diluted 10 times to the clotting time of the control buffer.

## Discussion

All commercial IVIG products are manufactured via modifications of the Cohn–Oncley cold ethanol fractionation process using cryo-poor pooled human plasma as the starting material. Following the recognition that contaminating thrombogenic factors in IVIG preparations could pose a risk to patients, regulatory agencies have required IVIG manufacturers to include steps proven to remove them.

Of the multiple coagulation factors and zymogens that could potentially pose a thrombogenic risk in IVIG preparations, FXI has long been recognized as the most serious (8–10). FXI is converted to its active form (FXIa) upon proteolysis by activated FXII (FXIIa), by thrombin, or by autoactivation (23). While the primary substrate of FXIa is FIX, FXIa has been shown to activate coagulation factors FX, FV, and FVIII, and inhibit the anticoagulant tissue factor pathway inhibitor (TFPI). These multiple points of impact of FXIa on the coagulation cascade may explain why even small quantities of residual FXIa can pose a risk in patients receiving IVIG therapy.

We have previously described the manufacturing process of a 5% maltose-stabilized IVIG and demonstrated that CEX chromatography was capable of reducing FXIa to undectable levels (18). The results of the current study are an important validation of this manufacturing step, confirming that it is a robust means of removing FXIa activity and antigen in a novel manufacturing process for a 10% glycine-stabilized product.

Results of the Western blot analysis performed on the intermediate and final products of the GC5107 manufacturing process are illustrative (Figure 2), demonstrating that while coagulation factors FII, FVII, FIX, and FX are effectively removed in the fractionation process in the Fraction I + III filtrate, FXI remains at a residual ratio of approximately 15%–16%, and is removed only after CEX chromatography. Due to its high isoelectric point (8.9–9.1) (14), FXIa is difficult to separate from IgG using ethanol precipitation alone. CEX chromatography utilizes a ceramic-based resin with 1.5 times higher binding capacity for IgG than standard resins. After washing and elution, Figure 2 demonstrates that the resulting IgG preparation was essentially free of FXI/FXIa.

These results were further quantified using ELISA (Figure 3). While fractionation steps result in a FXI log reduction factor of approximately 1.2 in the Fraction I + III supernatant, residual FXI persists in the subsequent intermediate products until after the CEX chromatography step, which resulted in a FXI log reduction factor of greater than 3.9. The entire manufacturing process resulted in a FXI log reduction factor of ≥5.4.

Because starting pools of human plasma can vary naturally in FXI/FXIa content, we further evaluated the robustness of the process using FXIa spiking studies (Figure 4), assessing FXI/FXIa antigen by ELISA and FXIa activity by TGA (Table 1). The results demonstrated that CEX chromatography reduced FXI/FXIa antigen to very low levels in samples spiked with 32 times and 158.3 times the concentration of that present in normal samples. FXIa activity in both spiked samples was below detection limits following CEX chromatography (<1.56 mIU/ml). Notably, the high IgG binding capacity of the CEX resin (>100.0 g/L) resulted in excellent IgG recovery (Table 1). This study confirms both the robustness and efficiency of FXIa removal by CEX chromatography.

Published studies have reported that several IVIG manufacturing processes, including pasteurization, octanoic acid fractionation, low pH treatment, and AEX chromatography are capable of reducing FXIa to below detection limits (15, 16). The spiking study described here is novel, however, as the capacity of the CEX chromatography step to remove FXIa was challenged with concentrations of FXIa up to five times higher than those found in cryo-poor plasma.

Wu and colleagues reported results of a study evaluating the removal of FXIa from an IG manufacturing process using the same CEX resin employed here (CM ceramic hyperD) (24). In this study, 1 IU/ml of FXIa was added to the IG supernatant obtained through caprylic acid precipitation. Conditional studies on FXIa removal from the IgG fraction using the CEX resin were then conducted. Under the reported study conditions, particularly in the elution pH range from 5.0–8.0, there was a tendency for IgG to co-purify with FXIa, demonstrating the challenge in separating IgG and FXIa using the CEX process.

However, under the novel CEX process conditions described here for GC5107 using the same resin but with an elution pH of 4.5, even in the presence of high concentration of FXIa, IgG and FXIa are effectively separated. The pH of the CEX loading buffer is 5.0, which is significantly lower than the pI of FXIa (8.9–9.1). Thus, positively charged FXIa strongly binds to the CEX resin and does not co-elute with IgG under elution conditions, but is instead stripped during the subsequent clean-in-place step.

Overall procoagulant activity in the final products of 9 commercial scale batches of GC5107 was assessed using the TGA, chromogenic FXIa and NaPTT assays. It has been demonstrated that NaPTT times longer than 200 s and NaPTT ratios of more than 0.8 in IVIG preparations are associated with low rates of TEEs (25). Our study revealed a NaPTT time longer than 250 s in all 9 batches of GC5107 and a ratio greater than or equal to 1.0 (Table 2). This analysis confirms both the presence of very low levels of FXIa and batch consistency.

These results, together with those of Park et al. (18), confirm that inclusion of a CEX chromatography step in the IVIG manufacturing process effectively removes FXIa antigen and reduces procoagulant activity in the final IVIG preparation to below detection limits. It is hoped that this new manufacturing process serves to further improve product safety.

## Data Availability

The original contributions presented in the study are included in the article, further inquiries can be directed to the corresponding author.
